# Intensity Modulated Proton Therapy with Advanced Planning Techniques in a Challenging Hepatocellular Carcinoma Patient

**DOI:** 10.7759/cureus.1674

**Published:** 2017-09-10

**Authors:** Smith Apisarnthanarax, Jatinder Saini, Avril O'Ryan-Blair, Jackie Castro, Stephen R Bowen

**Affiliations:** 1 Radiation Oncology, University of Washington/Seattle Cancer Care Alliance Proton Therapy Center; 2 Radiation Oncology, Seattle Cancer Care Alliance Proton Therapy Center; 3 Medical Dosimetry, Seattle Cancer Care Alliance Proton Therapy Center; 4 Radiation Oncology and Radiology, University of Washington School of Medicine

**Keywords:** intensity modulated proton therapy (impt), hepatocellular carcinoma, pencil beam scanning proton therapy, breath-hold, functional liver imaging

## Abstract

The use of radiation therapy has been increasing over recent years for the treatment of hepatocellular carcinoma (HCC). Proton beam therapy (PBT) has emerged as a promising treatment option for HCC patients due to its dosimetric advantages of sparing more normal liver tissue from radiation at low to moderate doses compared to photon-based treatments while still delivering high doses of radiation to tumors. The PBT therapy may be particularly beneficial in high-risk HCC cirrhotic patients with large, bulky tumors and/or vascular invasion complicated by surrounding perfusion abnormalities. We present a case of a 62-year-old male with an unresectable 13 cm diffusely infiltrative HCC tumor with main portal vein invasion and elevated alpha-feta protein (AFP) of 37,200 that was intolerant of standard sorafenib treatment. He was treated with hypofractionated PBT to 67.5 GyE in 15 fractions using a novel combination of simultaneously integrated boost intensity modulated proton therapy (SIB-IMPT), breath hold technique, and functional liver imaging with technetium-99m [^99m^Tc] sulfur colloid single-photon emission computed tomography (SPECT/CT) to assist in the differentiation of tumor and normal liver. He had a complete radiographic and biochemical response by AFP normalization by seven months post-treatment without evidence of radiation hepatotoxicity.

## Introduction

Hepatocellular carcinoma (HCC) is the most common primary malignancy arising from the liver and is the fifth most common malignancy worldwide [1]. The patients with unresectable HCC tumors treated with catheter-based therapies have a two-year overall rate of 30-65% and those with gross vascular tumor invasion have an extremely poor prognosis with a median survival of only two-three months if left untreated [2-3]. These patients, particularly those with large, bulky tumors, pose difficult challenges when treating with external beam therapy as the remaining non-tumor liver volume is limited and often already compromised due to cirrhosis. Radiation-related hepatotoxicity is omnipresent as a concern for potentially life-threatening complication. Proton beam therapy offers a promising radiation treatment modality to maximize liver sparing from radiation while delivering definitive high doses of radiation. We present a challenging case of a 62-year-old male with a large HCC and extensive vascular invasion successfully treated with definitive hypo-fractionated proton therapy using advanced techniques that integrated pencil-beam scanning intensity modulated proton therapy with a breath hold motion management and functional liver imaging.

## Case presentation

**Figure 1:**
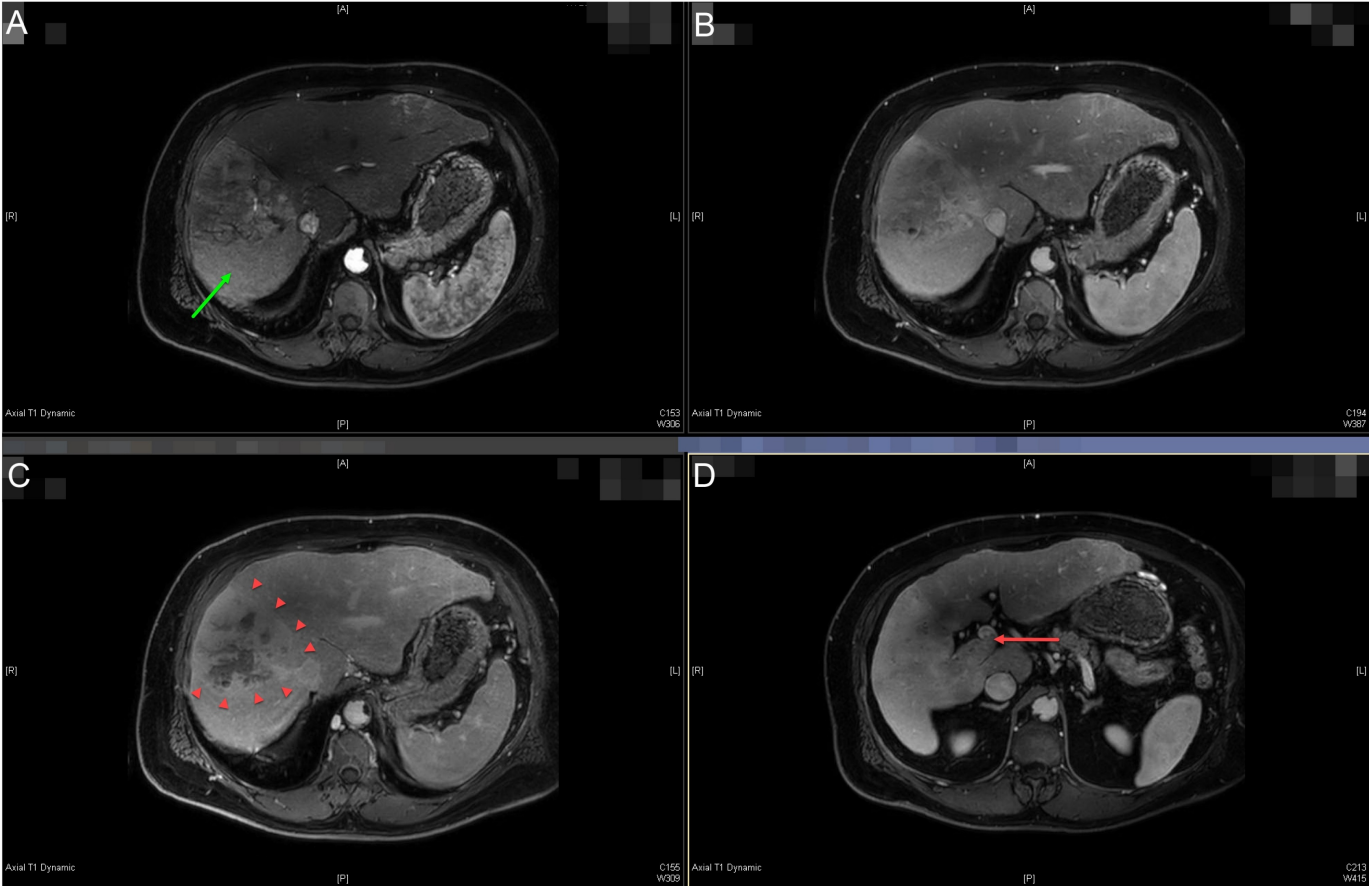
Pre-treatment diagnostic magnetic resonance imaging (MRI) of the abdominal scan. Multi-phase liver protocol MRI abdomen axial images showing arterial (A), venous (B), and delayed (C) phases of the diffusely infiltrative and heterogeneously enhancing hepatocellular carcinoma tumor involving segments five through eight (best seen on panel C, arrowheads). The portal vein tumor thrombus is seen extending into the main portal vein on venous delay (D - red arrow). Peri-tumoral perfusion abnormalities are seen in panel A (green arrow).

A 62-year-old Caucasian male with a history of successfully treated hepatitis C and Child-Pugh A5 cirrhosis presented with diffuse abdominal pain. Workup with ultrasound and follow-up multiphase abdominal computed tomography (CT) scan revealed a heterogeneously enhancing and infiltrative liver mass measuring approximately 10 x 9 x 13 cm involving segments five through eight suspicious for a hepatocellular carcinoma (HCC) with right portal vein tumor thrombus extending to the main portal vein through Liver Imaging Reporting and Data System (LI-RADS 5V tumor). The appearance of the tumor was complicated by prominent perfusion anomalies within segments five and eight. The magnetic resonance imaging (MRI) of the abdomen confirmed these findings (Figure 1), and the chest CT was negative for metastatic disease. Initial alpha-feta protein (AFP) was significantly elevated at 24,976.

 

No targetable lesion was seen on ultrasound due to the infiltrative nature of the lesion and CT-guided biopsy was performed but was nondiagnostic (fibrotic benign liver tissue). Despite the lack of tissue confirmation, the consensus of the multidisciplinary liver tumor board was to treat the patient for HCC, given the radiographic appearance, gross vascular invasion and elevated AFP in the setting of underlying hepatitis C and cirrhosis. The treatment with sorafenib 400 mg PO twice daily was recommended, which was complicated by severe fatigue, anorexia, and hyponatremia necessitating hospitalization. Sorafenib was restarted at 50% dose reduction after recovery from his hospitalization. Definitive external beam radiation therapy with hypofractionated proton beam therapy was recommended given the concern for the patient’s intolerance of sorafenib tolerance and the potential reduced tumor efficacy of continuing sorafenib at reduced doses. Proton therapy was chosen to limit the dose to the uninvolved normal liver and surrounding gastrointestinal organs considering the high tumor with the normal liver volume ratio. His pre-radiation AFP peaked at 37,200.

 

**Figure 2:**
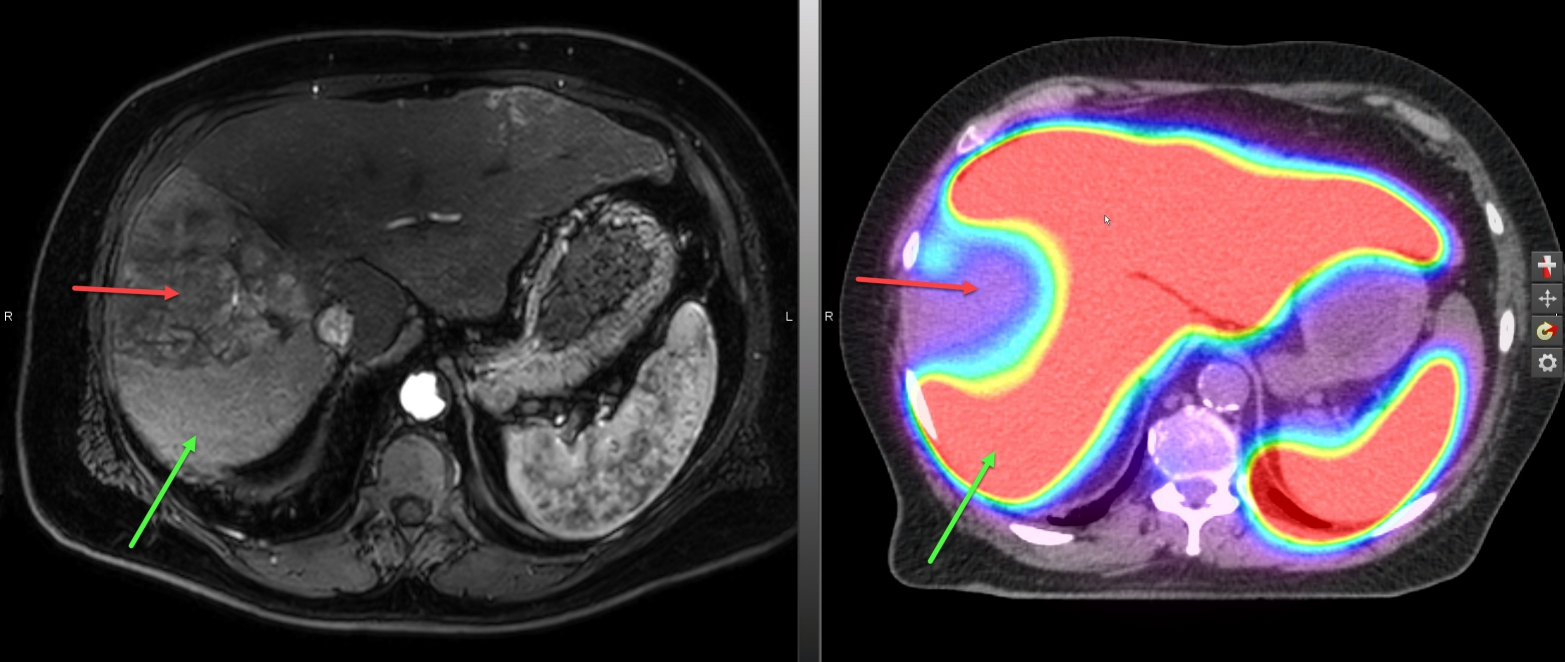
The treatment planning sulfur colloid single-photon emission computed tomography (SPECT/CT) scan. The technetium-99m [^99m^Tc] sulfur colloid SPECT/CT scan with which images sulfur colloid uptake in liver Kupffer cells and has been shown to correlate with global clinical liver function, was used to help identify and delineate both gross tumor and uninvolved normal liver. Areas of high sulfur colloid uptake represent non-GTV liver (red color wash) and the large well-defined photopenic defect (red arrow) corresponds to the tumor. The presumed perfusion abnormalities seen on the magnetic resonance imaging (MRI) scan (left panel) show high uptake of sulfur colloid on SPECT/CT (green arrows) and provides confirmation that these areas in the liver are unlikely infiltrated with tumor (red arrow).

Since fiducial markers were not able to be placed (tumor was not visible on ultrasound) for tumor motion assessment or image guidance, the patient was CT simulated in the supine position with both arms abducted above the head using the Active Breathing Coordinator (ABC)™ breath hold system (Elekta Instrument AB, Stockholm, Sweden) in the end-exhale position at 20% of the maximal deep inhalation volume. Multiple phases (non-contrast, arterial, venous, and delayed) scans were acquired. Considering the challenges in contouring the gross tumor volume (GTV) posed by the infiltrative nature of the tumor and perfusion abnormalities on imaging, a technetium-99m [99mTc] sulfur colloid single-photon emission computed tomography (SPECT/CT) scan was registered to the treatment planning CT and utilized to assist in the delineation of the gross tumor volume (GTV) as previously published by our group [4-5] (Figure 2).

 

Margins of 5 mm were added to the GTV to create a clinical target volume (CTV) and account for tumor margin uncertainties. The CTV was also extended 10 mm medial to the furthest extent of the main portal vein tumor thrombus to account for the proximal microscopic extension. Nonisotropic margins of 5 mm radially and 8 mm superiorly/inferiorly were then applied to the CTV and GTV to generate planning target volumes (PTV1 and PTV2, respectively).

**Figure 3:**
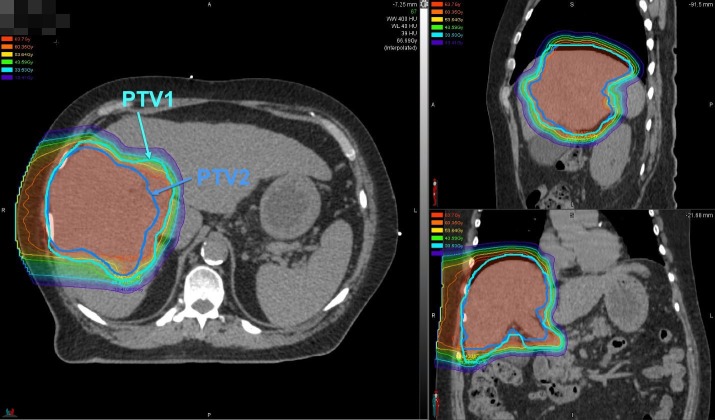
Intensity modulated proton therapy with simultaneous integrated boost. Intensity modulated proton therapy with simultaneous integrated boost pencil beam scanning to deliver 45 Gy relative biologic effectiveness (RBE) to planning target volumes (PTV1) (encompasses clinical target volume) (CTV) and 67.5 Gy (RBE) to PTV2 (encompasses gross tumor volume) (GTV) using a right lateral beam delivered twice with volumetric rescanning.

Intensity modulated proton therapy (IMPT) with a pencil beam scanning (PBS) was utilized to deliver 45 GyE to the PTV1 and a simultaneous integrated boost (SIB) of an additional 22.5 GyE to the PTV2 for a total prescribed dose of 67.5 GyE in 15 daily fractions (Figure 3).

**Figure 4:**
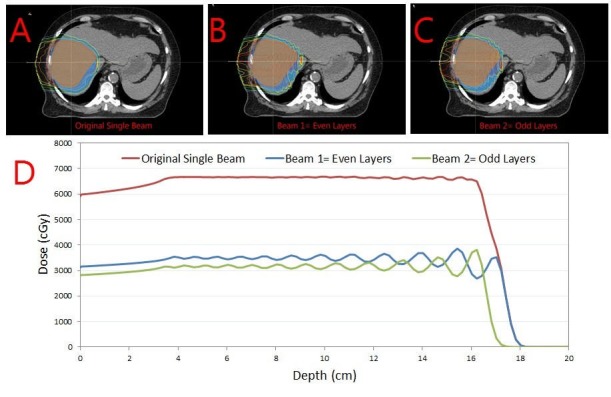
Alternating energy layer volumetric rescanning. Alternating energy layer volumetric rescanning technique (AELVR). The original single beam (A) is split into two beams using the AELVR technique, resulting into beams with even (B) and odd (C) layers. The one-dimensional dose distribution through the center of the target volume is shown in panel D.

A single right lateral beam angle was used with alternating energy layer volumetric rescanning (AELVR), an advanced technique to mitigate the interplay effect of spot scanning delivery and respiratory motion while reducing the beam delivery time to improve breath hold duty cycle (Figure 4).

AELVR splits the original beam into odd and even layered beams so that only half the dose is delivered to the target in each beam. As the numbers of layers are cut in half, so are the number of breath holds required for each beam. Thus, AELVR allows volumetric rescanning by requiring only half the breath holds and also allows a recovery breathing time for the patient in between beams. Mean dose to the liver minus GTV was 19.0 GyE. Daily image guidance was performed with kilovoltage KV to KV imaging aligning to bony spine and for the weekly quality assurance, slow CT scans were obtained to verify liver positioning during an end-exhaled breath hold.

**Figure 5:**
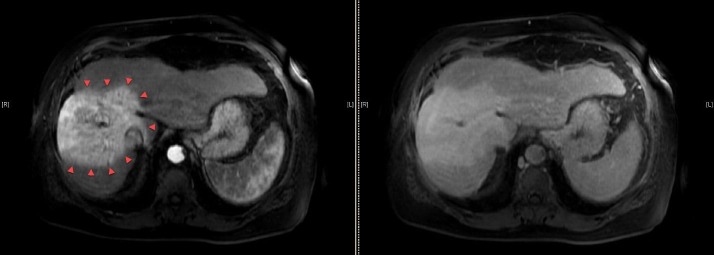
Post-radiation diagnostic magnetic resonance imaging (MRI) of the abdominal scan. The MRI abdominal scan axial images seven months after completion of proton therapy. Areas of hyperenhancement (red arrowheads) on arterial phase (left panel) correspond to planned dose distribution shown in Figure 3. No areas of washout are seen on delay phase (right panel), signifying a complete radiographic response.

He completed treatment with the expected side effects of mild fatigue and skin reaction. His AFP declined rapidly during treatment, reaching down to 7,800 upon completion of the treatment and subsequently normalizing to 6.6 at five months post-treatment. At this time, he developed severe chest wall pain in the irradiated area without evidence of rib fracture or tumor recurrence on imaging that required oral steroids and high-dose nonsteroidal anti-inflammatory drugs (NSAIDs) to control. Follow-up imaging at seven months post-treatment demonstrated a radiographically complete response in the primary tumor with evidence of post-radiation changes (Figure 5). As of the last follow-up, he has not developed any clinical evidence of hepatic decompensation with a stable Child-Pugh score of A5. 

## Discussion

In this report, we describe the first case of combining the use of IMPT with breath hold motion mitigation, functional liver imaging, and an advanced form of PBS volumetric rescanning. The use of each of these advanced techniques was necessary in addressing specific technical challenges posed by this case. 

Firstly, the absence of fiducial markers to serve as a tumor surrogate presented difficulty with tumor motion assessment and image guidance during treatment if free-breathing or abdominal compression treatment was being considered. The treatment during end-exhaled breath hold eliminates the need to account for tumor motion during simulation or treatment. However, combining breath hold treatment with PBS is more complex than with photon-based treatment. Since the treatment in PBS is delivered by scanning individual energy layers sequentially, the duration of beam delivery and in turn number of required breath holds is greatly dependent on the speed of the scanning magnets and the energy layer switching time. Whereas common scanning magnets are capable of delivering proton spots within an energy layer in less than one second, cyclotron-dependent energy layer switching in older systems such as the IBA Proteus Plus (IBA International, Louvain-La-Neuve, Belgium) is much slower over three to five seconds. This practically means that the beam delivery time is a strong function of the number of energy layers. For a typical locally advanced HCC patients, planned using PBS without constraining the number of energy layers, as per our experience, requires nearly eight to ten breath holds. Many patients have difficulty complying with these many breath holds in succession, particularly if there is time-sensitive pressure to relinquish the proton beamline to another room in a multi-room facility. In order to reduce the beam delivery time, and thereby reduce the number of required breath holds per beam, several strategies for energy layer number reduction are possible: (1) increasing the energy layer width using a ridge filter, (2) increasing the depth-spacing between energy layers, or (3) delivering alternating energy layers. These energy layer reduction strategies must be considered as a trade-off in increasing the target dose non-uniformity. The AELVR delivery technique utilized when treating the patient in this report effectively reduced the number of required breath holds to a manageable four per field while also mitigating the interplay effect through volumetric rescanning.

Secondly, the infiltrative nature of the primary tumor and surrounding perfusion abnormalities on imaging created uncertainty in delineating the borders of the GTV. To assist in reducing this uncertainty and improving the accuracy of distinguishing between tumor and normal liver, we obtained a [99mTc] sulfur colloid SPECT/CT scan and co-registered it to our treatment planning CT. Our group has previously published our experience in using this imaging modality as a tool for more accurately measuring total liver function and for potential functional liver avoidance treatment planning [4-5]. In this case, we used the sulfur colloid SPECT/CT scan to primarily confirm the areas considered to be perfusion abnormalities on diagnostic and treatment planning scans that were not involved with tumor (which are depicted as areas of photopenia).

Lastly, IMPT was utilized as a method to deliver dose painting (simultaneous integrated boost) of different PTVs: 45 GyE to cover the microscopic extension of tumor (CTV) and 67.5 GyE to treat the gross primary tumor and vascular involvement (GTV). The IMPT treatment was planned through use of single lateral beam angle as it presented in the most direct path to the tumor. The novel combination of these approaches resulted in a complete biochemical (AFP normalization) and radiographic response without a decline in hepatic liver function.

It should be noted that this treatment was not without toxicity as the patient did experience grade three chest wall toxicity as a result of 248 cc of chest wall receiving at least 60 GyE. Delivering high doses to the chest wall was not avoidable without compromising tumor coverage, given the size of his tumor and the peripheral location of the tumor adjacent to the right chest wall. In this patient, different beam angles would not have reduced the chest wall toxicity risk since the PTV substantially overlapped with the chest wall. In other patients, consideration of beam angles that do not overlap or range into the chest wall would be important to minimize chest wall toxicity. The use of PBS, as opposed to passive scattering techniques (e.g. uniform scanning) that would have resulted in higher proximal edge doses, was the only treatment planning modality that successfully minimized chest wall doses. As more data are gathered for chest wall dose constraints in these patients and incorporated into IMPT optimization, chest wall toxicity may be further minimized.

## Conclusions

The treatment of HCC tumors in cirrhotics patients with proton therapy is complex and presents several treatment challenges that need be addressed. This report describes the use of multiple advanced planning approaches with simultaneously integrated boost intensity modulated proton therapy (SIB-IMPT) that resulted in an excellent treatment outcome in a high-risk HCC patient. As the number of complex HCC cases that need external beam radiation therapy increases, the use of these advanced proton therapy techniques may be more common in place.
